# Included or excluded pelvis – does the inclusion of the pelvis in the scoliotic process influence the outcome?

**DOI:** 10.1186/1748-7161-7-S1-O46

**Published:** 2012-01-27

**Authors:** A Circo, C Coillard, C Rivard

**Affiliations:** 1 Sainte Justine Hospital, Montreal, Canada

## Background

Even though scoliosis is principally a deformity of the spine and the rib cage, some authors demonstrated that the pelvis can be included in the scoliotic process. Bernard Bricot concluded that the patients that presented with an excluded pelvis have a more evolving scoliosis. With regards to the pelvis we have two categories: the pelvis is excluded or included in the scoliotic process. In the first case the pelvis should be perfectly balanced, where as in the second group we can find a tilt, torsion or rotation of the pelvis.

The purpose of this retrospective cohort study was to verify if the inclusion of the pelvis has an influence for the outcome of the treatment.

## Materials and methods

Two groups where studied: first group composed of 77 patients that needed surgical treatment compared to the second group composed of 101 consecutive patients seen in the clinic and treated with SpineCor in 2003-2004 [1,2].

## Results

In the first group 16 out of 77 patients (20.8%) had an included pelvis compared to the control group where 22 out of 101 (21.8%) had an included pelvis. Most of the patients with an included pelvis in the control group had a thoraco-lumbar curve compared to the surgery group where the majority where thoracic and double curves.

## Conclusion

There is no difference in the outcome of the treatment of patients with included or excluded pelvis and it seems that regardless of the outcome, the same percentage of 21% of patients had an included pelvis.
